# Herpes Simplex Keratitis and Vitamin D Receptor Agonist: Two Case Reports

**DOI:** 10.3390/diseases13020038

**Published:** 2025-01-30

**Authors:** Atsushi Kawahara

**Affiliations:** Yoshida Eye Hospital, 2-31-8, Hondori, Hakodate 041-0851, Hokkaido, Japan; atsusi-k@coral.plala.or.jp; Tel.: +81-138-53-8311

**Keywords:** herpes simplex keratitis (HSK), herpes simplex virus, cornea, vitamin D, vitamin D receptor (VDR), calcitriol, medication adherence

## Abstract

Background: Herpes simplex keratitis (HSK) is a disease characterized by the recurrent infection of the cornea, mainly due to infection caused by herpes simplex virus type 1. The suppression of recurrence can suppress progressive corneal scarring, ulcers, and perforation. Cornea contains vitamin D receptors (VDRs). VDR agonists show antimicrobial activity. Case presentation: In this case report, I describe two female patients aged 76 and 85 years old in whom the administration of a VDR agonist led to the suppression of the recurrence of HSK. The former patient had repeated HSK recurrence for over 10 years after the initial infection. The latter patient developed HSK immediately after vitrectomy, and her cornea remained susceptible to infection, resulting in recurrence. Both patients were trying to suppress recurrence by applying acyclovir ophthalmic ointment, but their medication adherence was declining. So, they switched to oral treatment with 0.5 μg of the VDR agonist per day, and since then, there has been no recurrence of HSK. Oral treatment with the VDR agonist is still ongoing. Conclusions: This report highlights the cases where ways were examined to improve medication adherence in elderly patients who had a risk of HSK recurrence. Both patients responded to VDR agonist treatment and were able to suppress recurrence.

## 1. Introduction

Herpes simplex keratitis (HSK) occurs as a result of infection with herpes simplex virus type 1 or type 2, which are large viruses belonging to the human alpha-herpesvirus family [1]. Most cases are caused by herpes simplex virus type 1, a double-stranded DNA virus that is 152 Kbp in size [2]. The prevalence of herpes simplex virus type 1 infection in the world’s population aged 49 years or younger is approximately 67% [3]. Herpes simplex virus type 1 is a neurotropic virus that is transmitted through direct contact with mucous membranes and causes either no symptoms or transient epithelial keratitis [4]. After initial infection, herpes simplex virus type 1 is transported retrogradely along axons to the neuronal cell bodies of the trigeminal ganglion, and then it becomes latent in sensory neurons [5,6]. It is then reactivated, and the virions are transported along the axon to the ocular surface, where the active virions are released into the corneal tissue [7]. This repeated infection causes recurrent HSK, and 40% of patients experience 2 to 5 recurrences, while 11% experience 6 to 15 recurrences [8]. Corneal scarring caused by repeated recurrences has a significant impact on a patient’s visual function. HSK usually presents as a dendritic corneal epithelial ulcer with spherical ends. If left untreated or diagnosed late, it can progress to a geographic ulcer. The progression of corneal ulcers and the resulting corneal perforations are caused by the excessive degradation of the collagen in the corneal stroma. Collagen degradation is promoted by infectious microorganisms and corneal stromal keratocytes (fibroblasts). The reactivation of herpes simplex virus causes natural killer cells to produce cytokines and chemokines, which promote antiviral immune responses. Interleukin-8 produced by natural killer cells and herpes simplex virus-infected corneal epithelial cells promotes the influx of natural immune cells such as neutrophils. The stimulation provided to keratocytes from interleukin-1β produced by infiltrating neutrophils promotes the production of matrix metalloproteinases (particularly matrix metalloproteinase-2 and matrix metalloproteinase-9) in herpes simplex virus-infected corneal epithelial cells, leading to collagen degradation [9,10]. At this stage, microorganisms are no longer required for collagen degradation. The only treatment for the stromal melting of advanced corneal ulcers is steroids, and it becomes more difficult to stop the progression to perforation. Therefore, suppressing recurrence is important for HSK management, as treatment becomes difficult if recurrence occurs repeatedly or the condition persists for a long time. However, there are no clear guidelines for recurrence suppression [1].

Vitamin D receptors (VDRs) are present in corneal epithelial cells [11,12] and corneal stromal keratocytes [13]. Calcitriol is the most active form of vitamin D and is a VDR agonist. VDR agonists affect angiogenesis, antibacterial activity, and antiviral activity by activating a VDR, and as a result, they contribute to maintaining the barrier function of corneal epithelial cells and promoting corneal wound healing [14,15,16,17,18,19]. One study [20] showed the antiviral effects of VDR agonists on patients with COVID-19. It has also been reported that there is a negative correlation between the concentration of active vitamin D in the serum and the prevalence of HSV infection [21]. Therefore, VDR agonists have the potential to become HSK therapeutics.

Antiviral drugs such as acyclovir, ganciclovir, and trifluorothymidine are effective against HSK [1]. In particular, acyclovir is available in the form of ophthalmic ointment, oral medication, and intravenous medication. However, the long-term administration of oral or intravenous medication is not a realistic method for suppressing recurrence. In addition, there is concern that the application of ophthalmic ointment may lead to a decrease in medication adherence, particularly in the elderly. Therefore, in this report, I describe two cases of elderly patients with a high risk of HSK recurrence who were switched from acyclovir ophthalmic ointment to VDR agonist (calcitriol) administration as a method of suppressing recurrence.

## 2. Case Presentation 1

**Figure 1:**
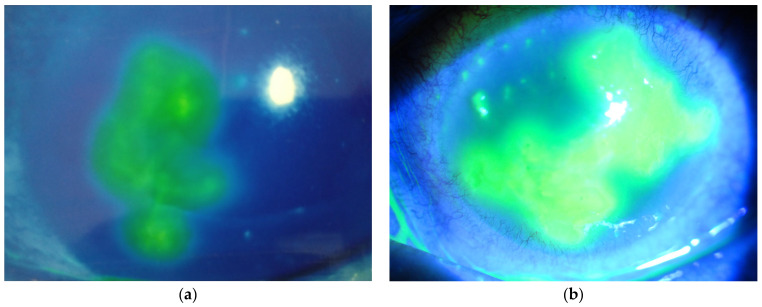
(**a**) shows a corneal staining photograph at the first onset of HSK, and (**b**) shows a staining photograph of recurrent HSK.

The first case was a 76-year-old female patient who had been under outpatient observation for about 20 years for intraocular lens-implanted eyes, allergic conjunctivitis, and dry eye disease. She was prescribed tranilast and sodium hyaluronate for both eyes as eye drops. During a visit to Yoshida Eye Hospital in 2010, she complained of blurred vision in the left eye, and slit-lamp microscopy revealed the first onset of HSK (Figure 1a). The intraocular pressure of the left eye was 20 mmHg, and there was no inflammation of the anterior chamber. The patient was instructed to discontinue the previously prescribed medication for the left eye and apply acyclovir ophthalmic ointment and levofloxacin eye drops. Levofloxacin was used to protect against mixed bacterial infections. After treatment began, the corneal ulcer gradually shrank and healed after 6 months, leaving behind corneal stromal opacity, so she was instructed to discontinue acyclovir and levofloxacin. During this period, the corrected visual acuity of the left eye decreased from 20/25 to 20/28. Five years later, the patient again noticed blurred vision in the left eye, and when examined, the recurrence of HSK was observed, and the visual acuity had decreased to 20/50. The intraocular pressure was 20 mmHg, and there was no anterior chamber inflammation. The same treatment as before was applied, and after 2 months, the ulcer had disappeared, and she was instructed to continue using acyclovir ophthalmic ointment and sodium hyaluronate eye drops to suppress recurrence. Acyclovir oral treatment was not applied because acyclovir and its metabolites, which are excreted by the kidneys, may damage the kidneys of patients [22] who are given long-term treatment. In particular, long-term treatment is not suitable for the elderly. Her visual acuity had recovered to 20/28. However, HSK recurred 1 year after the first recurrence. The visual acuity was 20/50, intraocular pressure was 56 mmHg, and anterior chamber inflammation was observed. In addition to the aforementioned treatment, treatment with fluorometholone eye drops, betamethasone subconjunctival injection, acyclovir oral administration, and intraocular pressure-lowering drugs (eye drops (brinzolamide and timolol), oral administration, and intravenous injection) was added. As the condition improved, the treatment was reduced, and after 3 months, HSK, high intraocular pressure, and anterior chamber inflammation disappeared, and the visual acuity recovered to 20/28. The patient was then again instructed to continue using acyclovir ophthalmic ointment and sodium hyaluronate eye drops to suppress recurrences. After that, there were similar recurrences three times before the latest recurrence, and the visual acuity during the quiescent period had decreased to 20/50 due to corneal scarring (stromal opacity). The latest recurrence was recognized during a checkup in January 2023. The visual acuity of the left eye had decreased to 20/200, and a slit-lamp microscope examination revealed a geographic ulcer (Figure 1b). The intraocular pressure was 21 mmHg, and there was no anterior chamber inflammation. Treatment was carried out in the same way as before, and after 6 months, visual acuity had recovered to 20/50, and the patient was instructed to continue using acyclovir ointment. During the follow-up period, I checked her recent adherence to the ophthalmic treatment. She confessed that she was having difficulty applying the acyclovir ointment and was self-suspending more frequently, and she requested an alternative treatment method.

This patient was receiving oral treatment for hypertension with nifedipine, dyslipidemia with pravastatin, chronic gastritis with lansoprazole, insomnia with brotizolam and ramelteon, and osteoporosis with risedronate. Her internal medicine doctor reported that her treatment progress and medication adherence were good. Risedronate inhibits bone resorption by inhibiting the function of osteoclasts, thereby suppressing bone turnover in patients with osteoporosis. Additionally, calcitriol, a VDR agonist, is also used to treat osteoporosis. It promotes the absorption of calcium in the intestinal tract and increases the serum calcium level by promoting the reabsorption of calcium in the kidneys, thereby activating osteoclasts and osteoblasts to improve bone metabolism and promote bone formation. Therefore, I suggested a change in treatment, focusing on the effects of VDR agonists on the cornea. She and her internal medicine doctor agreed to this, and the osteoporosis treatment was changed from risedronate to calcitriol, and acyclovir ointment was discontinued. Table 1 shows the changes in her blood test data for osteoporosis. From 1 month to 6 months after changing the treatment, the serum calcitriol (1,25-dihydroxycholecalciferol (D3)) level was higher than normal, and the calcium and phosphorus levels remained normal. Her ophthalmological treatment was switched back to using tranilast and sodium hyaluronate eye drops for both eyes, as before the onset of HSK. To date, HSK has not recurred, and only corneal stromal opacity remains (visual acuity 20/50). In addition, when she was asked about her medication adherence during follow-up, it had improved.

## 3. Case Presentation 2

**Figure 2:**
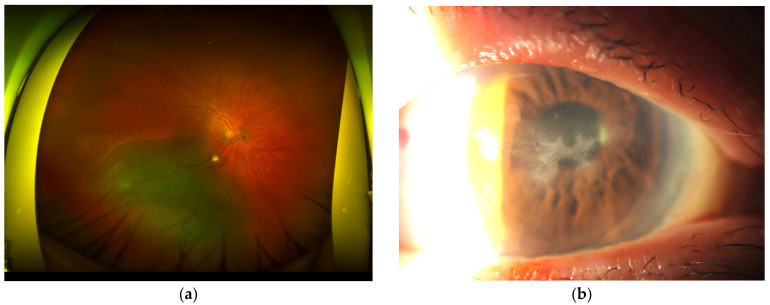

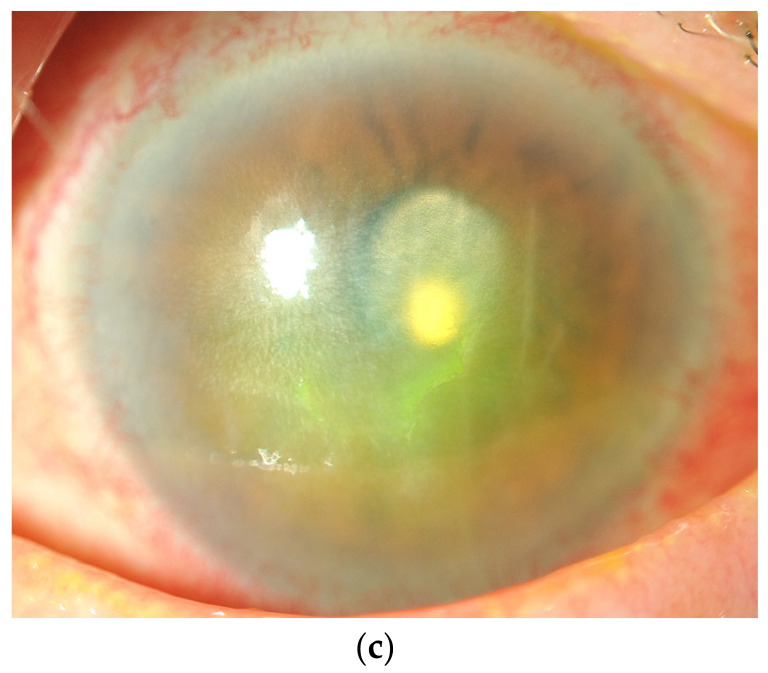
(**a**) shows a fundus photograph of a detached retina, (**b**) shows a photograph of the cornea at the first onset of HSK, and (**c**) shows a photograph of the cornea of recurrent HSK.

The second case was an 85-year-old female patient who was referred to Yoshida Eye Hospital in October 2023 for the treatment of a rhegmatogenous retinal detachment in her right eye. At the initial examination, a retinal tear was observed in the inferior peripheral area of the retina, and the retina was detached from this area to the macular area (Figure 2a). Both eyes were intraocular lens-implanted eyes, and the corrected visual acuity of the right eye was 20/400 (20/25 in the left eye). The patient was admitted to the hospital the day after the consultation and underwent a vitrectomy under local anesthesia (with intraocular tamponade using sulfur hexafluoride gas). After the surgery, the patient was placed in a face-down position and started treatment with eye drops. The eye drops were moxifloxacin, betamethasone, and bromfenac. However, at the 10-day follow-up examination, retinal re-detachment was observed in the area of the initial lesion. Vitrectomy (with scleral buckling and intraocular tamponade using octafluoropropane) was performed again the day after the next, and a face-down position and eye drop treatment were resumed. During the post-operative follow-up period, superficial punctate keratitis developed and gradually worsened, and the patient also complained of subjective symptoms related to the ocular surface, so this was then diagnosed as the development of post-operative dry eye disease, and sodium hyaluronate eye drops were added to the treatment regimen 10 days after the re-operation. As for the retinal lesion, there was no re-detachment even after the intraocular gas levels had decreased, so the requirement to maintain a face-down position was lifted 16 days after the re-operation. During her hospital stay, the retina was completely re-attached, but the condition of the cornea hardly improved. The superficial punctate keratitis in the central corneal region had worsened to corneal erosion, but it was judged that she could be observed as an outpatient, so she was discharged from the hospital. A total of 44 days after the second surgery, she visited the hospital as an outpatient. Her visual acuity in the right eye was 20/300, intraocular pressure was 16 mmHg, and there was mild anterior chamber inflammation, a re-attached retina, and macular edema. She also had subjective symptoms of epiphora, and slit-lamp microscopy revealed a dendritic corneal epithelial ulcer in the central corneal region (Figure 2b). She was diagnosed with HSK, and betamethasone and bromfenac eye drops were discontinued, and acyclovir ophthalmic ointment and oral medication (acyclovir) were added to the treatment (moxifloxacin and sodium hyaluronate eye drops were continued). She took the acyclovir oral medication for a week. A month after the change in treatment, the ulcer had healed with corneal stromal opacity remaining. The acyclovir ointment was discontinued, and anti-inflammatory eye drops (fluorometholone and bromfenac) were restarted to calm down the post-operative inflammation. However, 2 months later (4 months after the re-operation), she complained of pain and tearing in her right eye and visited the hospital. Her visual acuity was 20/2000, and slit-lamp microscopy revealed the recurrence of HSK (Figure 2c), so the anti-inflammatory eye drops were stopped, and acyclovir (ointment and oral medication) was added to the treatment, again. Consequently, HSK subsided a month after recurrence. At this time, anterior chamber inflammation was evaluated objectively for the first time using a laser flare meter (FC-700, Kowa Co., Ltd., Tokyo, Japan), and the flare value was 22.9 photon counts/ms, indicating that anterior chamber inflammation was still present (normal value: between 1.3 and 7.6 [23]). In addition, the central subfield thickness of the macular area was evaluated using optical coherence tomography on the same day, and a value of 590 μm was indicated, indicating significant macular edema. Therefore, a subtenon triamcinolone acetonide injection was administered. After the injection, I instructed her to resume betamethasone eye drops as an anti-inflammatory agent and to continue using acyclovir ophthalmic ointment to suppress the recurrence of HSK. I had her stop using moxifloxacin eye drops, as she had already been using them for about 5 months. Her treatment regimen consisted of betamethasone eye drops, sodium hyaluronate eye drops, and acyclovir ophthalmic ointment. During the subsequent follow-up, the flare value fluctuated between 11 and 13 photon counts/ms, and subtenon triamcinolone acetonide injection was added to treat the recurrence of macular edema. However, she confessed that she was exhausted from the treatment so far and that her medication adherence was declining, especially with regard to the application of acyclovir ophthalmic ointment, which was very difficult. I considered applying oral acyclovir treatment, but as mentioned in Case 1, long-term oral acyclovir treatment is problematic for the elderly, so it was not applied.

As for medications other than ocular medications, this patient was taking nifedipine for hypertension, atorvastatin for dyslipidemia, famotidine for chronic gastritis, brotizolam for insomnia, alogliptin for diabetes, and raloxifene for osteoporosis. The gastroenterologist reported that the patient’s treatment progress and medication adherence were good. Raloxifene exerts its effects via estrogen receptors. Its mechanism of action is that, after binding to estrogen receptors, it exerts an inhibitory effect on bone resorption similar to that of estrogen via cytokines involved in bone metabolism. Thus, I proposed that the osteoporosis treatment be changed to a VDR agonist (calcitriol) and that acyclovir ointment be discontinued. She and her treating physician agreed to this. As shown in Table 1, 1,25-D3 remained at a higher level than normal, and calcium remained at a normal level for the period up to 6 months after the change in treatment. Phosphorus levels were lower than normal only after 1 month but then remained normal. Since the start of calcitriol treatment, ophthalmic treatment for her right eye has been limited to two types of eye drops: betamethasone and sodium hyaluronate. Although anterior chamber inflammation and macular edema remain, HSK has not recurred since the change in treatment (visual acuity 20/200). In addition, her medication adherence has improved.

## 4. Discussion

Acyclovir is the most commonly used treatment for herpes simplex virus infections. Acyclovir is a prodrug that is phosphorylated by the herpes simplex virus protein thymidine kinase and host enzymes in host cells to form acyclovir triphosphate. This is the active form of acyclovir. Since thymidine kinase is of viral origin, the levels of the active form mainly increase in infected cells. The active form competes with deoxyguanosine trisphosphate and is incorporated into the viral genome during replication, causing premature termination [1]. In the treatment of HSK, ophthalmic ointment, oral medication, and intravenous acyclovir preparations are available. Normally, the application of ophthalmic ointment, which has the highest therapeutic effect, is chosen. The oral medication used as an adjuvant drug has the characteristic of having a bioavailability that is about eight times higher than that of intravenous administration, while the absorption rate decreases as the amount absorbed increases [24]. Valaciclovir, which is an ester-bonded valine to acyclovir used to improve the absorption rate, has a bioavailability equivalent to that of the intravenous formulation as an oral medication [25]. Therefore, oral medication is often chosen as an adjuvant drug for ophthalmic ointment. In this report, too, when treating HSK onset and recurrence, the patient was cured by using ophthalmic ointment and oral medication (valaciclovir) as auxiliary medication. However, acyclovir has weaknesses. These include drug resistance caused by virus strains that do not produce thymidine kinase or metabolize acyclovir and the fact that acyclovir is ineffective against latent herpes simplex virus because its mode of action consists of inhibiting viral replication. These weaknesses also become a problem when taking acyclovir orally for a long period of time to suppress HSK recurrence [1].

Case 1 had concurrent dry eye disease and was therefore susceptible to corneal infection and had experienced six recurrences since the initial occurrence of HSK. Case 2, who had diabetes, had lost strength due to two vitrectomies and the associated face-down positioning. After the surgery, she developed dry eye disease. During the follow-up period, anterior chamber inflammation and macular edema persisted, so she had to continue steroid treatment. These factors are thought to have led to the continued susceptibility of the cornea to infection, and even though she had only one case of HSK recurrence, there was still a sufficient risk of recurrence. In addition, Case 1 was 61 years old when she first developed HSK, but she was 76 years old at the time of the writing of this report. Case 2 was 84 years old when she underwent vitrectomy, and both patients were elderly. A major factor that reduces medication adherence in chronic ophthalmic diseases is advancing age [26]. In particular, since the main medication used to suppress recurrence in this report was an ophthalmic ointment, it is highly likely that the combination of ophthalmic ointment and eye drops would have further reduced medication adherence. In addition, the long-term administration of acyclovir may cause kidney damage [22]. Based on the above, it was necessary to find a way to improve medication adherence using a treatment other than acyclovir.

Calcitriol (1,25-D3), a VDR agonist, is an exocrine hormone with immune-boosting and anti-inflammatory effects that is secreted into the tear fluid from the lacrimal gland [16]. Several papers have reported the effects of calcitriol on the cornea. Lu et al. showed that calcitriol promotes corneal wound healing in mice [14]. Jabbehdari et al. demonstrated the wound healing effects of calcitriol in human corneal limbal epithelial cells and mouse corneas [27]. In this report, it was also shown that calcitriol reduces the expression of inflammatory molecules such as intercellular adhesion molecule 1, toll-like receptor 3, interleukin-6, interleukin-8, and TNF-alpha. Yin et al. showed that calcitriol improves corneal epithelial barrier function by increasing transepithelial resistance and decreasing inulin permeability [28]. Kumar et al. showed that calcitriol reduces herpes simplex virus type 1 viral load and mRNA expression in herpes simplex virus type 1-infected HeLa cells [29]. These reports suggest the potential of calcitriol as a treatment for HSK. In addition, if the serum calcium and phosphorus levels can be adequately controlled, long-term administration, which is considered a weakness of acyclovir oral medication, will be possible, and there is the possibility that calcitriol can be used as a recurrence suppressant.

The patients in this report had poor adherence to topical ophthalmic medications but good adherence to oral medications taken for conditions other than ophthalmology. In addition, because the patients had osteoporosis, the osteoporosis treatment medication was changed to calcitriol in an attempt to elicit a recurrence suppression effect on HSK. The serum calcium and phosphorus levels remained normal, and although follow-up is necessary in the future, the change in treatment this time was successful in suppressing the recurrence of HSK. However, there is a limitation to this report. That is, the patients in this report had osteoporosis, so they were eligible for calcitriol treatment. Calcitriol is not indicated for HSK at present. Vitamin D supplements are an alternative for patients who are not indicated for treatment, but further research is needed to determine whether supplements are effective for HSK. In addition, randomized controlled trials and other research are needed to generalize the use of calcitriol as a treatment for HSK.

## 5. Conclusions

Suppressing HSK recurrence is one of the important factors in maintaining visual function in elderly patients. However, there are no clear guidelines for suppressing recurrence. In present cases, the treatment plan for elderly patients with corneal susceptibility to infection was initially to use acyclovir ophthalmic ointment regularly to prevent the recurrence of HSK. However, because the patients had low medication adherence, the risk of recurrence was high, and it was necessary to change the treatment method. Considering that the patients were being treated for osteoporosis with another type of oral medication other than the VDR agonist, the osteoporosis medication was changed to the VDR agonist (0.5 μg per day). This change in treatment led to an improvement in medication adherence and was able to suppress HSK recurrence. These two case reports show the potential usefulness of the VDR agonist as an adjuvant drug for HSK treatment and as a recurrence suppressant, and further research, such as randomized controlled trials, is expected in the future.

## Figures and Tables

**Table 1:** Blood test data for osteoporosis.

Item	After Starting Calcitriol	Case 1	Case 2	Normal Level
1,25-D3 (pg/mL)	1 M	93.8	86.4	20–60
	3 M	94.3	78.2	
	6 M	92.6	83.1	
Calcium (mg/dL)	1 M	9.5	9.1	8.6–10.1
	3 M	9.6	9.0	
	6 M	9.4	9.3	
Phosphorus (mg/dL)	1 M	2.9	2.3	2.5–4.5
	3 M	2.6	2.5	
	6 M	3.2	3.1	

1,25-D3: 1,25-dihydroxycholecalciferol; M: month.

## Data Availability

The data are not publicly available since they containing information that could compromise the privacy of research participants.

## References

[1] Sibley D., Larkin D.F.P. (2020). Update on Herpes simplex keratitis management. Eye.

[2] Gatherer D., Depledge D.P., Hartley C.A., Szpara M.L., Vaz P.K., Benkő M., Brandt C.R., Bryant N.A., Dastjerdi A., Doszpoly A. (2021). ICTV Virus Taxonomy Profile: Herpesviridae 2021. J. Gen. Virol..

[3] James C., Harfouche M., Welton N.J., Turner K.M., Abu-Raddad L.J., Gottlieb S.L., Looker K.J. (2020). Herpes simplex virus: Global infection prevalence and incidence estimates, 2016. Bull. World Health Organ..

[4] Akhtar J., Tiwari V., Oh M.J., Kovacs M., Jani A., Kovacs S.K., Valyi-Nagy T., Shukla D. (2008). HVEM and nectin-1 are the major mediators of herpes simplex virus 1 (HSV-1) entry into human conjunctival epithelium. Investig. Ophthalmol. Vis. Sci..

[5] Kropp K.A., Sun G., Viejo-Borbolla A. (2023). Colonization of peripheral ganglia by herpes simplex virus type 1 and 2. Curr. Opin. Virol..

[6] Wang S., Song X., Rajewski A., Santiskulvong C., Ghiasi H. (2023). Stacking the odds: Multiple sites for HSV-1 latency. Sci. Adv..

[7] Rowe A.M., St Leger A.J., Jeon S., Dhaliwal D.K., Knickelbein J.E., Hendricks R.L. (2013). Herpes keratitis. Prog. Retin. Eye Res..

[8] Wishart M.S., Darougar S., Viswalingam N.D. (1987). Recurrent herpes simplex virus ocular infection: Epidemiological and clinical features. Br. J. Ophthalmol..

[9] Nishida T., Sugioka K., Fukuda K., Murakami J. (2021). Pivotal Role of Corneal Fibroblasts in Progression to Corneal Ulcer in Bacterial Keratitis. Int. J. Mol. Sci..

[10] García-López C., Rodríguez-Calvo-de-Mora M., Borroni D., Sánchez-González J.M., Romano V., Rocha-de-Lossada C. (2023). The role of matrix metalloproteinases in infectious corneal ulcers. Surv. Ophthalmol..

[11] Dai Y., Zhang J., Xiang J., Li Y., Wu D., Xu J. (2019). Calcitriol inhibits ROS-NLRP3-IL-1beta signaling axis via activation of Nrf2-antioxidant signaling in hyperosmotic stress stimulated human corneal epithelial cells. Redox Biol..

[12] Panigrahi T., D’Souza S., Shetty R., Padmanabhan Nair A., Ghosh A., Jacob Remington Nelson E., Ghosh A., Sethu S. (2021). Genistein-Calcitriol Mitigates Hyperosmotic Stress-Induced TonEBP, CFTR Dysfunction, VDR Degradation and Inflammation in Dry Eye Disease. Clin. Transl. Sci..

[13] Lu X., Chen Z., Watsky M.A. (2021). Effects of 1,25 and 24,25 Vitamin D on Corneal Fibroblast VDR and Vitamin D Metabolizing and Catabolizing Enzymes. Curr. Eye Res..

[14] Lu X., Chen Z., Lu J., Watsky M. (2023). Effects of Topical 1,25 and 24,25 Vitamin D on Diabetic, Vitamin D Deficient and Vitamin D Receptor Knockout Mouse Corneal Wound Healing. Biomolecules.

[15] Gorimanipalli B., Shetty R., Sethu S., Khamar P. (2023). Vitamin D and eye: Current evidence and practice guidelines. Indian J. Ophthalmol..

[16] Lu X., Elizondo R.A., Nielsen R., Christensen E.I., Yang J., Hammock B.D., Watsky M.A. (2015). Vitamin D in Tear Fluid. Investig. Ophthalmol. Vis. Sci..

[17] Rolando M., Barabino S. (2023). Dry Eye Disease: What Is the Role of Vitamin D?. Int. J. Mol. Sci..

[18] Gombart A.F., Borregaard N., Koeffler H.P. (2005). Human cathelicidin antimicrobial peptide (CAMP) gene is a direct target of the vitamin D receptor and is strongly up-regulated in myeloid cells by 1,25-dihydroxyvitamin D3. FASEB J..

[19] Yuk J.M., Shin D.M., Lee H.M., Yang C.S., Jin H.S., Kim K.K., Lee Z.W., Lee S.H., Kim J.M., Jo E.K. (2009). Vitamin D3 induces autophagy in human monocytes/macrophages via cathelicidin. Cell Host Microbe.

[20] Entrenas Castillo M., Entrenas Costa L.M., Vaquero Barrios J.M., Alcalá Díaz J.F., López Miranda J., Bouillon R., Quesada Gomez J.M. (2020). Effect of calcifediol treatment and best available therapy versus best available therapy on intensive care unit admission and mortality among patients hospitalized for COVID-19: A pilot randomized clinical study. J. Steroid Biochem. Mol. Biol..

[21] Huang J., Wu Y., Wang M., Lin S. (2023). The association between serum 25-hydroxyvitamin D and the prevalence of herpes simplex virus. The association between serum 25-hydroxyvitamin D and the prevalence of herpes simplex virus. J. Med. Virol..

[22] Becker B.N., Fall P., Hall C., Milam D., Leonard J., Glick A., Schulman G. (1993). Rapidly progressive acute renal failure due to acyclovir: Case report and review of the literature. Am. J. Kidney Dis..

[23] Tugal-Tutkun I., Herbort C.P. (2010). Laser flare photometry: A noninvasive, objective, and quantitative method to measure intraocular inflammation. Int. Ophthalmol..

[24] Kaye S., Choudhary A. (2006). Herpes simplex keratitis. Prog. Retin. Eye Res..

[25] MacDougall C., Guglielmo B.J. (2004). Pharmacokinetics of valaciclovir. J. Antimicrob. Chemother..

[26] Negese Kebede B., Mohammed Seid S., Kefyalew B., Gesese E. (2024). Glaucoma medication non-adherence rate and associated barriers among glaucoma patients in Hawassa, Ethiopia. BMC Ophthalmol..

[27] Jabbehdari S., Yazdanpanah G., Chen E., Afsharkhamseh N., Ghassemi M., Anwar K.N., Fonteh C., Djalilian A.R., Kang K.B. (2021). Dose-dependent therapeutic effects of topical 1,25 OH-vitamin D3 on corneal wound healing. Mol. Biol. Rep..

[28] Yin Z., Pintea V., Lin Y., Hammock B.D., Watsky M.A. (2011). Vitamin D enhances corneal epithelial barrier function. Investig. Ophthalmol. Vis. Sci..

[29] Kumar A., Singh M.P., Kumar R.S., Ratho R.K. (2018). 25-Hydroxyvitamin D3 and 1,25 Dihydroxyvitamin D3 as an Antiviral and Immunomodulator Against Herpes Simplex Virus-1 Infection in HeLa Cells. Viral Immunol..

